# Review of Methods Suitable for Environmental Surveillance of *Salmonella* Typhi and Paratyphi

**DOI:** 10.1093/cid/ciaa487

**Published:** 2020-07-09

**Authors:** Graciela Matrajt, Lorraine Lillis, J Scott Meschke

**Affiliations:** 1 Department of Environmental and Occupational Health Sciences, School of Public Health, University of Washington, Seattle, Washington, USA; 2 PATH, Seattle, Washington, USA

**Keywords:** *Salmonella* Typhi, *Salmonella* Paratyphi, environmental surveillance, sampling

## Abstract

Typhoid fever is an enteric disease caused by the pathogens *Salmonella* Typhi and *Salmonella* Paratyphi. Clinical surveillance networks are lacking in many affected areas, thus presenting a need to understand transmission and population prevalence. Environmental surveillance (ES) has been suggested as a potentially effective method in the absence of (or in supplement to) clinical surveillance. This review summarizes methods identified in the literature for sampling and detection of typhoidal *Salmonella* from environmental samples including drinking water, wastewater, irrigation water, and surface waters. Methods described use a trap or grab sampling approach combined with various selective culture and molecular methods. The level to which the performance of identified methods is characterized for ES in the literature is variable, thus arguing for the optimization and standardization of ES techniques.

There is renewed interest in environmental surveillance (ES), as a programmatic tool, in the public health fight against *Salmonella enterica* serovars Typhi (*S.* Typhi) and Paratyphi (*S.* Paratyphi). Typhoid and paratyphoid fevers are enteric diseases and, as such, are shed fecally. Environmental surveillance of wastewater samples has been proven to be a highly effective tool in combating other enteric pathogens, particularly polio. This review summarizes typhoid and paratyphoid fevers as a public health concern and recounts methods that have been reported in the literature for sampling and detection of typhoidal *Salmonella* from environmental samples. Additionally, this review discusses particular use cases (ie, actual scenarios when such methods could be applied), for programmatic surveillance of *S*. Typhi and *S.* Paratyphi. Literature searches in PubMed and Web of Science were performed. Studies reporting on environmental surveillance on *S*. Typhi and *S.* Paratyphi included in this review were identified using combinations of the keywords typhoid fever, environmental surveillance, *S*. Typhi, *S.* Paratyphi, *Salmonella* spp, wastewater, sewage, septage, and water sampling.

*Salmonella* Typhi and *S.* Paratyphi are human-specific gram-negative bacterial pathogens, which are primarily responsible for typhoid fever and paratyphoid fever, respectively. Both are human-restricted pathogens (ie, they have no known animal reservoir), and are transmitted from person to person through the fecal–oral route by ingesting contaminated food or water, or by contact with fecal matter from acute or chronically infected individuals [1]. *Salmonella* Typhi represents a major human health risk in many parts of the world, especially in developing countries where there is open defecation [2], where fecal matter collection and disposal is inefficient or reused in agriculture [3], and where there is a lack of access to safe water [4]. In particular, typhoid fever is an endemic disease in several South Asian and sub-Saharan African countries. Outbreaks can occur even in endemic settings if an environmental variable, population immunity, or circulating strain characteristics shift [1, 5].

The current disease burden estimate is quite imprecise because often the reported numbers are based on cases that were severe and needed hospitalization, but most patients do not develop severe symptoms and are treated by local medical practitioners or remain untreated [5]. Knowing and acknowledging the disease burden is crucial for making informed public health decisions such as vaccine strategies, allocating resources, and monitoring the effects of interventions [6]. However, as the burden of typhoid fever greatly varies over space and time (ie, the incidence can vary within a single city or geographical area), localized clinical surveillance data may not be easily extrapolated [6]. Furthermore, traditional population-based study approaches to assess disease burden are resource intensive and expensive, requiring both robust laboratory infrastructure and population-based clinical data collection encompassing a substantial numbers of participants [7].

Given that typhoid fever is a disease that can transition between an active outbreak and a more latent/dormant endemic form [6], clinical surveillance alone may be inadequate for disease monitoring within a population. In such cases or in the absence of clinical surveillance, ES may be an effective tool.

## ENVIRONMENTAL SURVEILLANCE

Environmental surveillance refers to the collection and analysis of environmental samples (eg, drinking water, wastewater) for a specific pathogen or screening for indicators of fecal contamination. It is a practical tool for the protection of public health. For example, analysis of coliforms or other indicators in drinking water is routinely performed to assess water quality and ensure that public health is protected. ES of pathogens in water samples can be also useful for determining transmission pathways, identifying risk factors in outbreaks settings, and providing insight on the circulation of those pathogens within a population. The most developed ES program currently implemented for an enteric pathogen is for poliovirus (PV). ES is an integral part of the eradication effort and is used to guide PV vaccination campaigns in endemic areas, areas with a risk PV importation, or outbreak locations, and to monitor circulation of the virus in the population in the absence of clinical acute flaccid paralysis cases [8, 9]. Like PV, *S*. Typhi and *S*. Paratyphi may be shed asymptomatically in a population at high levels. In contrast to PV, typhoid fever is not nearing eradication and does not currently have a widely implemented routine vaccination program. As a result, the use cases for typhoid ES may deviate somewhat from that of PV ES. Still, the main principle behind ES for typhoid remains that as *S.* Typhi are fecally shed, its presence should be expected in the wastewater of a burdened population; thus, surveillance measures would inform on the disease burden in that population. Potential use cases identified for ES of typhoid include (1) a guide to support introduction of routine vaccination; (2) monitoring of intervention strategies (eg, vaccine or water, sanitation, and hygiene); and (3) surveys of emergence and transmission of *S*. Typhi into nonendemic regions or during outbreaks in endemic regions.

Given the ill-defined burden of typhoid fever, the primary use case for ES of typhoid is to guide vaccine deployment efforts, specifically for the new typhoid conjugate vaccines recently developed. ES can provide insight on country needs and thus the potential size of the vaccine market. This, in turn, would aid countries in their applications to Gavi, the Vaccine Alliance (Gavi) for support to implement a typhoid conjugate vaccine program by providing data on the burden of disease.

Additionally, ES may help identify risks of typhoid fever before an outbreak occurs by identifying the presence of asymptomatic carriers, in the same way as it has been used to identify asymptomatic carriers of polio, which in turn has played a role in preventing outbreaks [10–12]. ES has been used multiple times to determine the source of a typhoid fever infection, which has helped local authorities with decision making including banning/closing water sources such as water for irrigation [13] or consumption [14].

Despite the potential of using ES for *S*. Typhi and *S.* Paratyphi, programmatic surveillance networks have not yet been widely adopted. Rather, surveillance efforts have focused on blood culture or clinical diagnosis, yet these too are limited. ES for *S*. Typhi and *S.* Paratyphi has been successfully implemented in a variety of research studies.

## STUDIES INVOLVING ENVIRONMENTAL SURVEILLANCE FOR TYPHOID

### Assessing Risk Factors of Infection

A study conducted in Nepal [9], where typhoid fever is endemic, investigated drinking water sources geographically close to an identified typhoid fever hotspot to assess the public water quality. Ten different water sources were sampled weekly during 1 year. The samples were subjected to chemical, bacteriological, and molecular analyses to determine the pathogen risks. DNA of *S*. Typhi and *S*. Paratyphi A was detected in every water source sampled. High rainfall was identified as a key driver of such contamination.

Another study conducted in an endemic area of Congo combined a questionnaire and microbiological analyses of water samples from various sources to determine the possible presence of typhoid hotspots and the disease transmission route [15]. Water samples were collected from 3 military camps, as well as from the nearby general populations, and were tested for the presence of *S*. Typhi. The military camps were determined to be the likely *S*. Typhi hotspots, and proximity of these camps may have been a risk factor for disseminating *S*. Typhi to the general population.

An study in India [16] investigated > 1000 water samples obtained from a variety of sources to determine whether *S*. Typhi was resistant to antibiotics. More than 96 different strains of *S.* Typhi were identified from all of the sources sampled, most of which were resistant to antibiotics, thus supporting contaminated water sources as a risk factor.

A study conducted in Nigeria investigated various drinking water sources to assess the quality of the water and the risks for waterborne diseases [17]. Water was sampled from areas with a high number of waterborne cases reported and areas with a low numbers of cases. *Vibrio cholerae*, *S.* Typhi, and *Shigella dysenteriae* were found in most samples and it was suggested that drinking water sources had been contaminated during the heavy rain season by runoff of contaminated water.

### Identifying the Source of an Outbreak

Several studies were conducted in India during the massive typhoid fever outbreaks of December 1975–February 1976 [5], November–December 1995 [18], and 2014 [19] to determine the sources of the sudden outbreaks. In all of the studies, chemical and bacteriological analyses of municipal water from various sources (wastewater effluents, drinking wells and storage tanks, and water stored in the households) were performed. In all cases the municipal water was *S*. Typhi positive, and this was caused by repeated contamination of the water. The findings indicated that the chlorination methods in use in these settings was inadequate, resulting in extensive *S.* Typhi contamination, which in turn increased the incidence of cases. Contributors to contamination included heavy rains and leakages in the sewage drainage system, which caused inflow of contaminated water into the water supply systems.

A study conducted in Nepal during the typhoid fever outbreak of 2002 [20] concurrently screened blood samples of patients and municipal water for the presence of *S.* Typhi. All water isolates were positive for *S*. Typhi and showed an analytical similarity of 96%–100% to blood culture results, indicating that the municipal water was highly contaminated with *S*. Typhi.

Similarly, a study conducted in Pakistan [21] investigated food samples, water samples from wells and households, and stool samples from patients during a 2004 outbreak. *Salmonella* Typhi was found in 100% of well water samples, 65% of household water samples, 2% of food items, and 22% of clinical stool samples.

A subsequent study conducted in Pakistan during the 2016–2017 ceftriaxone-resistant *S*. Typhi outbreak [22] investigated drinking water samples from households and community water sources to identify disease risk factors. DNA of *S*. Typhi was detected in 22% of the water samples analyzed. Most cases were clustered around sewage lines. The epidemic curve indicated a propagated epidemic, suggesting continuous contamination of water sources, perhaps through mixing of sewage water with municipal water.

## ENVIRONMENTAL SURVEILLANCE METHODS FOR *S*. TYPHI

The methods currently used for collection of ES samples for *S*. Typhi or *S.* Paratyphi include either grab sampling of water using a variety of devices, or trap sampling using Moore swabs. These samples are then typically concentrated, enriched, and analyzed by culture or polymerase chain reaction (PCR)–based methods. Culture-based methods frequently use an enrichment step prior to plating on selective media, thus making direct enumeration of the *S*. Typhi or *S.* Paratyphi in the samples problematic, instead yielding a presence/absence result. Nonetheless in some cases, a most probable number approach may be used for enumeration. Colony morphology on selective and differential media is commonly used to determine a presumptive positive. Isolated colonies are then typically confirmed by agglutination and biochemical methods, serotyping, or molecular methods. For PCR-based approaches, DNA is extracted directly from the water samples, sample concentrates, enrichments, or isolated colonies, then subjected to PCR amplification.

### Grab Sampling Methods

Several grab sampling techniques have been described for collecting water for *S*. Typhi and *S*. Paratyphi screening, including via bottles, buckets, or directly from outlet pipes. Sampling of water typically tries to avoid sediments from the bottom and is often performed in duplicate or triplicate [15, 17, 23, 24]. The sample volume collected varies from several milliliters to several liters. Water is then transferred to sterile plastic (Nalgene) containers [23, 25], sterile glass bottles [7, 17, 26–28], sterile Abba-type (stainless steel) bottles [24], glass sample cells [29], or sterile WhirlPak bags [29, 30] and transported to a laboratory. Samples are typically stored in a cooler on ice or kept at 4°C during transport and until processing (usually within 48 hours) [3, 23–27, 29–34]. Physicochemical characteristics of the water sampled are also generally collected, including temperature, pH, conductivity, salinity, and dissolved oxygen [9, 17, 23–26, 29, 30, 32, 35–37]. Some environmental studies collect large volumes of water (several liters) and sample concentration is performed prior to analysis. Concentration is important to improve the sensitivity of detection by increasing the portion of the sample analyzed. In some studies, dead-end ultrafiltration using REXEED 25S ultrafilters have been used to concentrate large-volume (20 L) samples of water [29]. In many environmental studies, small-volume samples (50–100 mL) are filtered through a low-porosity filter membrane (eg, 0.45-μm nitrocellulose membrane filters), to collect and concentrate the pathogens before plating on culture media [38]. Other studies have used prebaked 0.7-μm-pore-size glass fiber filters to concentrate environmental samples [27]. It should be noted that filter-based concentration methods may be subject to clogging that varies with the sample matrix.

### Trap Sampling With Moore Swabs

In several studies, water or sewage has been collected using the Moore cotton tampon method [39]. In the original method, 40 × 40-mm cotton tampons were submerged on steel wire in water for 4–6 days, while modified versions of the method use pieces of pipe filled with rolled cotton gauze or folded gauze swabs. The method traps bacteria as water passes through the pipe or by the swab [40]. The recovered tampons/swabs are then sent to the laboratory inside sterile jars and subjected to enrichment culture for *S.* Typhi [41–44]. In addition to sewage sampling, Moore swabs have been used to sample river waters contaminated with sewage [45].

The sensitivity and reliability of the Moore swab technique has been evaluated by placing swabs in small sewers draining the homes of known *S*. Typhi carriers [46]. It was found that the sensitivity depends on the size of the sewer (the smaller the diameter of the sewer sampled, the better the sensitivity) and the number of swabs (sensitivity increases with an increasing number of swabs) [44, 46]. Additionally, random sampling of larger sewers with Moore swabs is not a sensitive approach [47]. It should be noted that trap-based sampling is inherently nonvolumetric and can only be quantified based on the time deployed. The affinity of the swab material for *S*. Typhi and *S.* Paratyphi has also not been thoroughly characterized under controlled conditions.

### Detection Methods

*Salmonella* Typhi and *S.* Paratyphi are generally recognized as fastidious organisms and are difficult to culture [7]. Still, several culture-based analytical protocols have been described for *S.* Typhi in drinking water, including methods by the United States Environmental Protection Agency (EPA) [48] and Public Health England (PHE) [49]. The EPA method was adapted from clinical microbiological methods and from methods for the analysis of food [49]. The method involves a general preenrichment step followed by a selective enrichment in a most probable number format, with positive tubes plated on 2 selective culture media (bismuth sulfite agar and Miller-Mallinson agar). Isolated typical colonies are then confirmed by agglutination and biochemical methods. The PHE method [49] was developed for the food production environment and generally targets salmonellae, but includes additional specific selective enrichment media (selenite cysteine broth) and selective solid media (xylene lysine desoxycholate agar, brilliant green agar, and Hynes deoxycholate citrate agar) for targeting *S*. Typhi and *S.* Paratyphi. It is worth noting that some media for salmonellae generally may be inhibitory for *S*. Typhi and *S.* Paratyphi. Furthermore, some studies have reported that culture media can favor the growth of *S*. Typhi while failing to grow *S*. Paratyphi B and vice versa [50]. Both the EPA and PHE methods use selenite cysteine broth for selective enrichment, though selenite broth has also been used to identify *S*. Typhi from sewage samples recovered using Moore swabs [43, 44]. Section 9260B of the Standard Methods for Examination of Water and Wastewater [51] also summarizes basic approaches for detection and characterization of salmonellae, including *S.* Typhi and *S.* Paratyphi, though many of the methods listed contain cautions on the limitation of the methods performance for detection of *S*. Typhi and *S.* Paratyphi. Despite several culture methods having demonstrated the ability to detect *S*. Typhi and *S.* Paratyphi in water or wastewater samples under some circumstances, no one culture method has demonstrated adequate reliability for programmatic ES.

PCR is a reliable tool to indicate the presence of typhoidal *Salmonella* DNA in water [7, 15]. A real-time quantitative PCR method originally developed for biological samples [52] has become the dominant, gold-standard method for the detection of *S.* Typhi in water samples [53]. However, PCR does not prove that viable bacteria are present in the water [7]. A variety of other PCR protocols have also been described for detection of *S*. Typhi, but none have yet demonstrated adequate breadth of detection and sensitivity for broad adoption in ES. Some studies have developed multiplex PCR methods or parallel methods for multiple targets to increase breadth and specificity of detection of *S*. Typhi and *S.* Paratyphi [54]. For example, primers targeting the *fliC-d* (phase-1 flagellin gene for d antigen H:d of *Salmonella enterica* serovar *S*. Typhi), *tyv* (tyvelose epimerase), and *viaB* (Vi antigen) have been described [29]. Quantification is possible using standard curves containing an *S.* Typhi genomic DNA standard [29, 53]. It should be noted that matrix-associated inhibition is a significant concern when using molecular methods for ES, especially for wastewater. Inhibition can lead to false-negative results and underestimates of the quantity of *S*. Typhi or *S.* Paratyphi present.

## CONCLUSIONS

Environmental surveillance is an important tool in the fight against typhoid fever. ES can determine the presence of *S.* Typhi and *S.* Paratyphi in environmental samples and provide insight on the circulation of the bacteria in asymptomatic populations. Several approaches for sampling and detection of *S.* Typhi and *S.* Paratyphi in environmental samples have been described, including grab- and trap-based sampling coupled with both culture and molecular methods, though each of these methods can lead to false-positive or false-negative results. As a reliable standard method is still lacking, an accurate and precise detection method for ES is needed. ES has the potential to supplement clinical blood surveillance or, in the absence of clinical surveillance, to serve as a marker of typhoid prevalence in a population, thus facilitating efficient deployment of vaccination campaigns.

## Notes

***Financial support.***This supplement is funded with support from the Coalition against Typhoid Secretariat, housed at the Sabin Vaccine Institute in Washington, DC and made possible by a grant from the Bill & Melinda Gates Foundation.

***Supplement sponsorship.*** This work was sponsored by Bill & Melinda Gates Foundation, OPP1182032.

***Potential conflicts of interest*.** The authors: No reported conflicts of interest. All authors have submitted the ICMJE Form for Disclosure of Potential Conflicts of Interest.
